# 175. Concordance of UTI Antibiotic Treatments with Facility-Specific Antibiograms in Nursing Homes

**DOI:** 10.1093/ofid/ofae631.055

**Published:** 2025-01-29

**Authors:** Madeline C Langenstroer, Sally Jolles, Christopher J Crnich, Robin Jump, Brigid Wilson, Taissa A Bej, Corinne Kowal, Oteshia Hicks, Lindsay Taylor, Grace Multhauf, Jon P Furuno, Evita T Santos, Hailey Shilling, Michelle H Zhou, David A Nace, Theresa N Murillo, Krishnendu Mangal

**Affiliations:** University of Wisconsin School of Medicine and Public Health, Saint George, UT; University of Wisconsin School of Medicine and Public Health, Saint George, UT; University of Wisconsin School of Medicine and Public Health, Saint George, UT; VA Northeast Ohio Healthcare System, Cleveland, Ohio; VA Northeast Ohio Healthcare System, Cleveland, Ohio; Louis Stokes Cleveland VA Medical Center, Cleveland, Ohio; Louis Stokes Cleveland VA Medical Center, Cleveland, Ohio; VA Northeast Ohio Healthcare System, Cleveland, Ohio; University of Wisconsin School of Medicine and Public Health, Saint George, UT; University of Wisconsin School of Medicine and Public Health, Saint George, UT; Oregon State University, Portland, Oregon; Yale New Haven Hospital, New Haven, Connecticut; Oregon Health and Science University, Portland, Oregon; Oregon State University College of Pharmacy, Portland, Oregon; University of Pittsburgh, Pittsburgh, Pennsylvania; UPMC Senior Communities, Sarver, Pennsylvania; UPMC Senior Communities, Sarver, Pennsylvania

## Abstract

**Background:**

Empiric antibiotic treatment of urinary tract infections (UTI) in nursing homes (NHs) is complicated by increasing antibiotic resistance. Antibiograms may enhance empiric antibiotic treatment decisions in this setting. We conducted a pilot study of a urine-specific antibiogram in 9 NHs in Oregon and Pennsylvania. The objectives of this study were to (1) characterize patterns of antibiotic resistance among uropathogens recovered from study NHs and (2) determine the concordance between antibiotic prescriptions and facility-specific antibiograms.

**Table 1.**
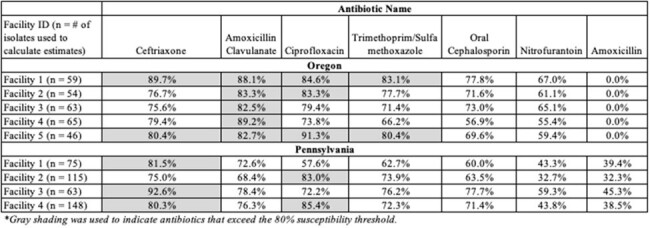
WISCA-Concordant Antibiotics across 9 NHs

**Methods:**

We developed a facility-specific weighted incidence syndromic cumulative antibiogram (WISCA) using two years of urine culture results for each study NH. The WISCA conveyed the cumulative susceptibility of facility-collected uropathogens to 7 antibiotics in three susceptibility strata ( >90%, 80-89%, and < 80%). Data on antibiotic prescriptions for the empiric treatment of UTI during the 6-month pilot were abstracted from facility health records and entered into a REDCap database. Antibiotic prescriptions with a probability of activity exceeding the 80% susceptibility threshold were classified as WISCA-concordant.

**Figure 1.**
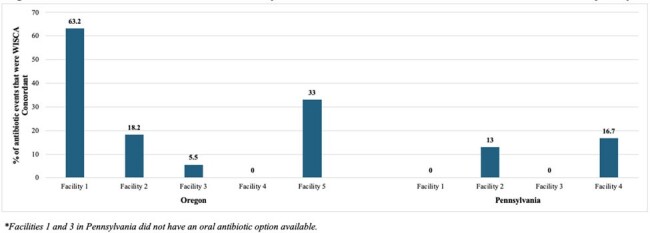
Concordance of Empiric Antibiotic Selection with Facility-Specific WISCAs

**Results:**

WISCA susceptibility patterns varied considerably across NHs and regions (Table 1). On average, two oral antibiotic options with projected activity exceeding 80% were available in most Oregon NHs. In contrast, oral antibiotic options with activity exceeding 80% were uncommon in Pennsylvania NHs. Ceftriaxone and Ciprofloxacin were the only antibiotics with activity in these facilities. There were 173 empiric antibiotic treatments for UTI in study NHs during the pilot. A minority of the antibiotics chosen were concordant with the facility-specific WISCA (n = 69, 39.9%), although this varied by facility (Figure 1).

**Conclusion:**

Our study demonstrates wide variability in antibiotic susceptibility of uropathogens at both the facility and regional levels. Antibiotics selected for the empiric treatment of UTI in study NHs were projected to have limited activity based on facility-specific WISCAs. Further studies to examine the factors influencing antibiotic choice and level of concordance with patient-specific culture results are ongoing.

**Disclosures:**

**Robin Jump, MD, PhD**, Abacus: Grant/Research Support|Merck: Grant/Research Support|Pfizer: Advisor/Consultant|Pfizer: Grant/Research Support **Jon P. Furuno, PhD**, Merck & Co., Inc: Grant/Research Support

